# Developing an *in vitro* validated 3D *in silico* internal carotid artery sidewall aneurysm model

**DOI:** 10.3389/fphys.2022.1024590

**Published:** 2022-12-20

**Authors:** Hang Yi, Zifeng Yang, Mark Johnson, Luke Bramlage, Bryan Ludwig

**Affiliations:** ^1^ Department of Mechanical and Materials Engineering, Wright State University, Dayton, OH, United States; ^2^ Boonshoft School of Medicine, Wright State University, Dayton, OH, United States; ^3^ Division of NeuroInterventional Surgery, Department of Neurology, Wright State University/Premier Health—Clinical Neuroscience Institute, Dayton, OH, United States

**Keywords:** internal carotid artery sidewall aneurysm (ICASA), hemodynamic behaviors, particle image velocimetry (PIV), computational fluid dynamics (CFD), flow field

## Abstract

**Introduction:** Direct quantification of hemodynamic factors applied to a cerebral aneurysm (CA) remains inaccessible due to the lack of technologies to measure the flow field within an aneurysm precisely. This study aimed to develop an *in vitro* validated 3D *in silico* patient-specific internal carotid artery sidewall aneurysm (ICASA) model which can be used to investigate hemodynamic factors on the CA pathophysiology.

**Methods:** The validated ICASA model was developed by quantifying and comparing the flow field using particle image velocimetry (PIV) measurements and computational fluid dynamics (CFD) simulations. Specifically, the flow field characteristics, i.e., blood flowrates, normalized velocity profiles, flow streamlines, and vortex locations, have been compared at representative time instants in a cardiac pulsatile period in two designated regions of the ICASA model, respectively. One region is in the internal carotid artery (ICA) inlet close to the aneurysm sac, the other is across the middle of the aneurysmal sac.

**Results and Discussion:** The results indicated that the developed computational fluid dynamics model presents good agreements with the results from the parallel particle image velocimetry and flowrate measurements, with relative differences smaller than 0.33% in volumetric flow rate in the ICA and relative errors smaller than 9.52% in averaged velocities in the complex aneurysmal sac. However, small differences between CFD and PIV in the near wall regions were observed due to the factors of slight differences in the 3D printed model, light reflection and refraction near arterial walls, and flow waveform uncertainties. The validated model not only can be further employed to investigate hemodynamic factors on the cerebral aneurysm pathophysiology statistically, but also provides a typical model and guidance for other professionals to evaluate the hemodynamic effects on cerebral aneurysms.

## 1 Introduction

Arterial walls involved with the bifurcated arteries within the circle of Willis have potential risks to form cerebral/intracranial aneurysms by abnormal focal enlargements, which may cause severe consequences due to intracranial hemorrhage into the subarachnoid space once the aneurysm sac is ruptured (Linn et al., 1996; Winn et al., 2002; Kaminogo et al., 2003; Jabbarli et al., 2016). Nearly 5% of the population in the United States have at least one cerebral aneurysm, and about 0.2% of them rupture annually (de Rooij et al., 2007; Sadasivan et al., 2013). However, there is an inadequate understanding of rupture risks when evaluating unruptured brain aneurysm discovered incidentally. How aneurysms grow and when aneurysm sac(s) will rupture is still ambiguous, due to the lack of fundamental studies on the pathophysiology of cerebral aneurysms (CAs) (Meng et al., 2014). Thus, to offer effective treatments more responsibly for intracranial aneurysms and improve patient experience, a systematic understanding of the pathophysiology of CAs is extremely important for clinicians.

Over past decades, many review publications summarized the efforts in studying hemodynamic factors on the pathophysiology of CAs, including varying risks associated with aneurysmal sac locations, high risk aneurysm morphologies, pre- and post-treatment states, and arterial blood flow conditions *in vivo*, *in vitro*, and *in silico* (Sforza et al., 2009b; Nieuwkamp et al., 2009; Nixon et al., 2010; Aoki and Nishimura, 2011; Jeong and Rhee, 2012; Fennell et al., 2016; Sheikh et al., 2020; Yu et al., 2021; Yi et al., 2022a). Although transcranial Doppler velocimetry (TDV), as a non-invasive *in vivo* manner, can be used to assess cerebral mean blood flow velocity in the cerebral arteries (Aaslid et al., 1982; Hart and Haluszkiewicz, 2000; Conde-Agudelo et al., 2015), this approach can only obtain values in limited local regions of the cerebral arteries rather than a thorough blood flow distribution in the arteries. Another *in vivo* method, phase-contrast magnetic resonance imaging (PC-MRI) has been used for blood velocity measurements, but it suffers from the relatively poor resolution, which can be an important limitation with respect to the small dimensions commonly encountered within CAs.

Alternatively, with the advantages of high image resolutions and in a time-resolved manner, particle image velocimetry (PIV) methods and their derivatives are increasingly used to measure blood flow patterns for *in vitro* CAs (Adrian, 1991; Kosugi et al., 2004; Nishino et al., 2004; Bouillot et al., 2014; Brindise et al., 2016; Hosseini et al., 2021). Yagi (2007) (Yagi et al., 2011; Yagi et al., 2013) employed the fluorescent scanning stereoscopic PIV to study flow impingement in a patient-specific internal carotid artery sidewall aneurysm (ICASA) from a transient flow regime and found the hydrodynamic instability of shear layer should not be neglected even at a low Reynolds number. Medero et al. (2020) found tomographic PIV has the feasibility to assess 4D flow MRI with high repeatability in the measurements of time-resolved and time-averaged velocity flow fields in a patient-specific intracranial aneurysm model. However, it also has limitations in measuring blood flow distributions in CAs using PIV, which are due to: 1) the known/unknown differences between the *in vitro* setups and *in vivo* patient-specific conditions, 2) unavoidable interpolation discrepancies and relatively low operational flexibilities, and 3) insufficient ability to capture the blood flow patterns near arterial wall, which is essential for wall shear stress (WSS) estimations. To address the above-mentioned deficiencies, computational fluid dynamics (CFD) based *in silico* methods in an accessible and non-invasive manner have been employed widely to predict the blood flow patterns in CAs, with the advantages of using physiologically based reconstructed models and initial/boundary conditions which can aid in identifying major translational knowledge gaps and provide a platform for implementing and evaluating potential solutions (Burleson et al., 1995; Kerber et al., 1999; Hongo et al., 2001; Jou et al., 2003; Steinman et al., 2003; Shojima et al., 2004; Alastruey et al., 2007; Sforza et al., 2009b; a; Botti et al., 2018; Soldozy et al., 2019; Rayz and Cohen-Gadol, 2020).

However, to the best of our knowledge, the majority of these studies solely used CFD without *in vivo* and/or *in vitro* verification and validation, influencing the acceptance of such simulations results. This technology remains limited within the clinical community, as they employed strong modeling assumptions (i.e., non-patient-specific assumptions) as well as varying solution strategies. More specifically, CFD results can be varied significantly among different research groups, although geometry, initial and boundary conditions, and blood properties were similar and only the solution strategies had to be individually adapted. Thus, the validation is one mandatory step for CFD modeling blood flow patterns in CAs. Several CFD studies have investigated the pathophysiology of CAs, in collaboration with the experimental tests (Ford et al., 2008; Raschi et al., 2012; Brindise et al., 2019). However, the above-mentioned studies considered the blood flow as a fully viscous laminar regime or a non-pulsatile flow condition, which may cause significant errors to estimate essential parameters within CAs.

**FIGURE 1:**
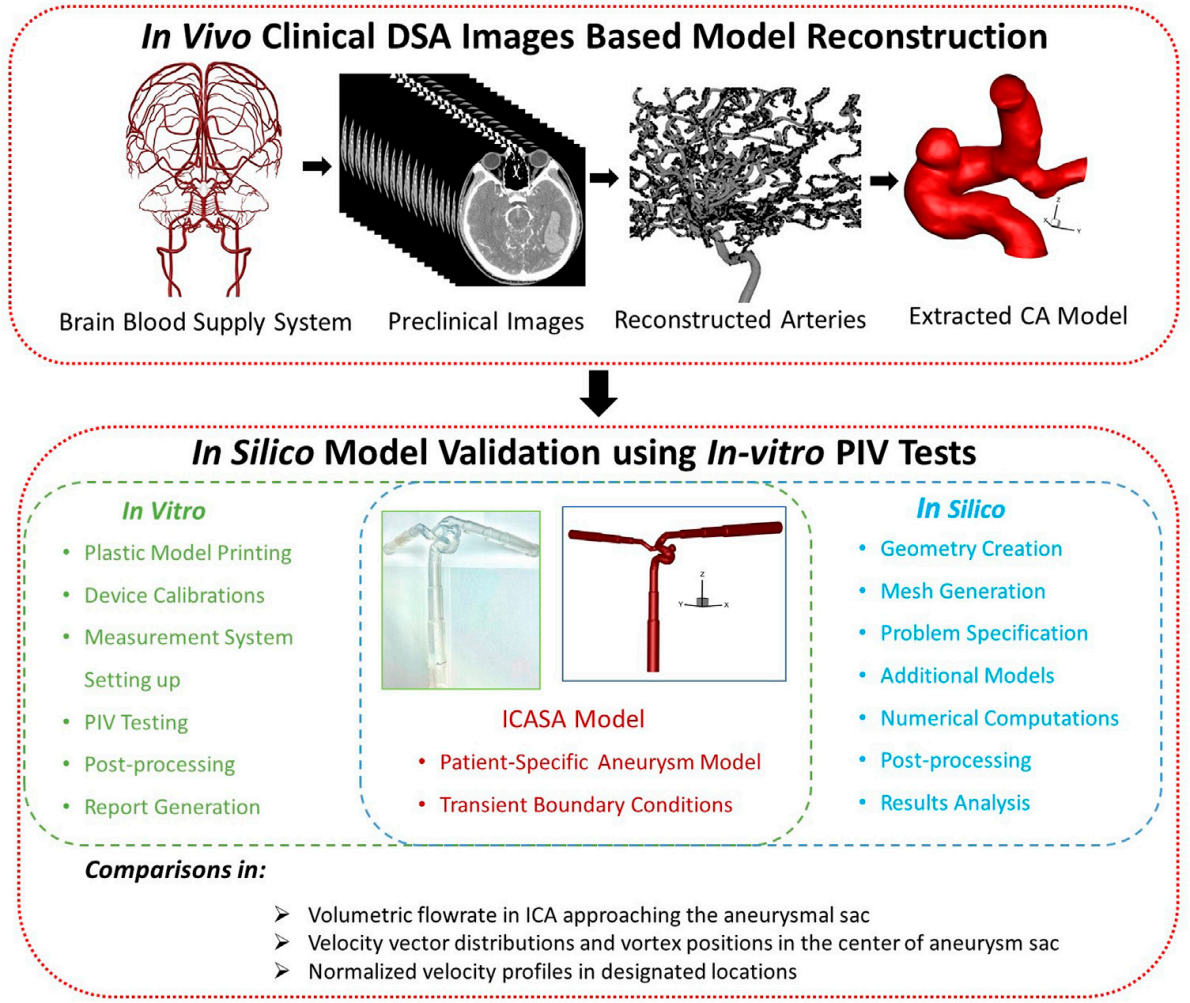
The framework of *in vitro* validated *in silico* study in the ICASA model.

To partially address the above-mentioned concerns, the objective of this study was to develop an *in vitro* validated *in silico* model (see Figure 1), which can be further employed to investigate hemodynamic characteristics such as WSS and oscillatory shear index (OSI) which have a significant impact on the CA pathobiology with statistical techniques. Specifically, the experimental validated CFD model was built by comparing the agreements of flow characteristics (i.e., blood flow rate, normalized magnitude of velocity, vectors distributions, flow streamlines, and vortex’s location) between PIV measurements and CFD simulations under the cardiac pulsatile flow conditions in an ICASA model. In addition, this study provided a benchmarked pathway for other researchers to design PIV experiments and conduct CFD simulations which are associated to the pathobiology of cardiovascular and neurovascular diseases.

## 2 Experimental settings

### 2.1 Aneurysmal replica

**FIGURE 2:**
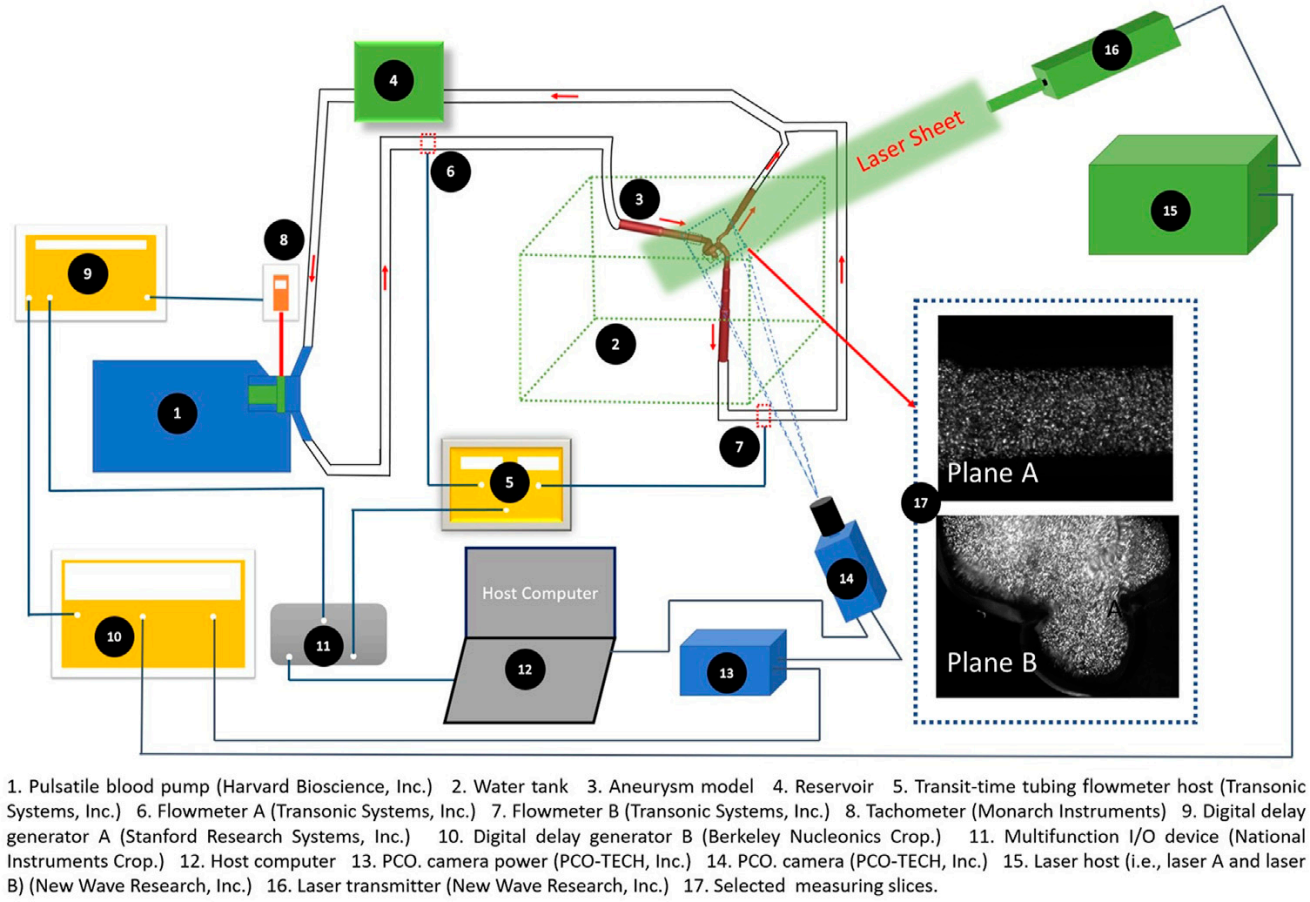
Experimental setup for PIV measurements.

A physical aneurysmal model (i.e., 70–80 years old, female, consent form was not required for de-identified patient data for the current research as approved by the Institutional Review Board (IRB) of Wright State University) was produced with a scaling factor of 3 to allow high-quality PIV measurements on the flow field inside (see Figure 1), with a minimum and maximum diameter of ∼6.4 and ∼15.5 mm of the arteries, respectively. Specifically, the aneurysm model was based on non-invasive 3D rotational angiographic images using Artis Zee systems (Siemens Medical Solutions USA, Inc., PA, United States), which were provided through a long-term collaboration with a hospital in Dayton (OH, United States). The physical hard plastic ICASA model was printed with WaterShed XC 11122 materials using a prototype machine at Proto Labs, Inc. (MN, United States) (see Figure 1 and Label 3 in Figure 2), in which the layer line was removed, and clear coat was applied on the interior surfaces. The exterior surfaces were finished with grit blasting. The WaterShed XC 11122 material is a translucent colorless stereolithography plastic that behaves similarly to acrylonitrile butadiene styrene to facilitate PIV investigations. The tolerance in the X/Y direction is ±0.05 mm and in the Z direction is ±0.125 mm, where X/Y are in-plane coordinates and Z is the out-plane coordinate associated with the printing process.

### 2.2 *In vitro* cerebral artery circulation

In accordance with the identical Reynolds number in the cerebral artery system with the real size, the mean volumetric flowrate in the PIV measurements is set as ∼ 2,084 ml/min, based on the findings in a previous study that the mean volumetric blood flow rate in the human ICA is about 306 ± 396 ml/min, with assumed 99.7% confidence interval (Oktar et al., 2006). As shown in Figure 2, a phantom circulation network was constructed to mimic cerebral arteries using artificial components, i.e., a Harvard Apparatus Pulsatile Blood Pump (Harvard Bioscience, Inc., MA, United States) (see Label 1 in Figure 2), a laboratory-made reservoir (see Label 4 in Figure 2), the plastic ICASA model (see Label 3 in Figure 2), and compliant plastic tubing. A heart rate was set as 60 ± 0.5 beats per minute in the Harvard Apparatus Pulsatile Blood Pump. The patient-specific transient pulsatile blood flow rates at the ICA (i.e., flow inlet) and ICA distal (i.e., one of flow outlets) were measured by the real time ultrasound flowmeter system (TS410 module and ME10 PXN inline sensors, Transonic Systems, Inc., NY, United States) (see Label 5, 6, and 7 in Figure 2). The flowmeter system has an absolute uncertainty of ±4% of the reading, with an ultrasound frequency of 1.8 MHz. Integrating with the user defined LabVIEW code (National Instruments Crop., TX, United States), one set of multifunction I/O device (USB-6218, National Instruments Crop., TX, United States) (see Label 11 in Figure 2) was employed to ensure that the inlet and outlet flow rates can be recorded simultaneously. The working fluid, i.e., water, was seeded with fluorescent polymer particles (PFFs) (10–45 μm) for PIV measurements.

### 2.3 PIV technique

A digital PIV system, including a Nd: Yag laser system (NewWave Gemini 120, New Wave Research, Inc., CA, United States) (see Label 15 and 16 in Figure 2), a high-resolution charge-coupled device (CCD) camera (PCO1600, PCO-TECH, Inc., Germany) (see Label 13 and 14 in Figure 2), the digital delay generator A (DG535, Stanford Research Systems, Inc., CA, United States) (see Label 9 in Figure 2), and the digital delay generator B (Model 575, Berkeley Nucleonics Crop., CA, United States) (see Label 10 in Figure 2), was used to accomplish the detailed flow field measurements. Two pulses of 120 mJ at the wavelength of 532 nm of the laser (12 Hz) were shaped into a sheet by a set of optics to illuminate the flow field. The camera was synchronized to capture two particle images corresponding to the two laser pulses. The laser sheet had a measured thickness of ∼2 mm through the region of interest to mitigate error associated with the out-of-plane movement. Seeding of the flow was accomplished with red-fluorescent micro-spheres of diameters between 10 and 45 μm. The test rig has the capability to make “phase-locked” measurements, which is important to capture the temporal feature of the intravascular flow. A laser tachometer (PLT200, Monarch Instruments, NH, United States) (see Label 8 in Figure 2) was used to detect the position of the pulsatile pump piston, which is directly related to the timing of the heart cycle. The pulse signal generated in the tachometer is used to trigger the whole PIV system. By adding a time delay into the pulse signal through the digital delay generator A, the pulsatile flow feature was “frozen” at the designated time instants. Therefore, the phase-averaged flow measurements can be obtained at different time instants within a cycle to quantify the temporal flow behaviors. The measurement was conducted under the well-controlled pulse rate (i.e., 60 ± 0.5 Hz) and volumetric flow rate. The water bath (see Label 2 in Figure 2) was used to enhance the visibility and control the fluid temperature to be the ambient room temperature (i.e., 295 ± 0.5 K).

### 2.4 PIV measurements

**FIGURE 3:**
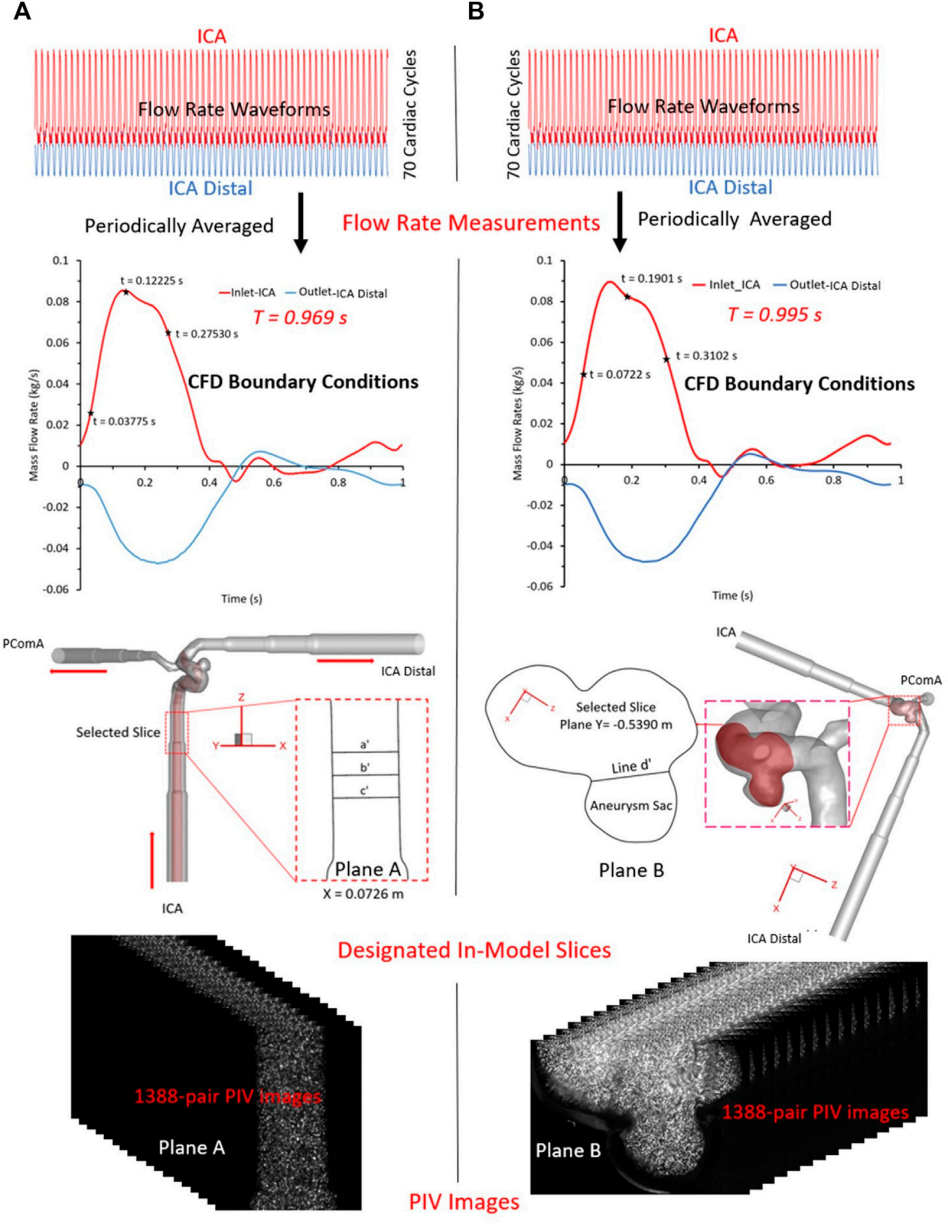
PIV measurements in the two selected planes: **(A)** plane X = 0.0726 m, and **(B)** plane Y = −0.5390 m.

Two representative cut-planes, i.e., plane A and plane B (see Label 17 in Figures 2, 3) were designated for the PIV measurements, i.e., plane A located in the ICA just upstream of the aneurysm sac, and plane B across the aneurysm sac in the middle. Prior to PIV tests, the camera was inspected to confirm it was facing perpendicularly to the designated cut-plane (see Figure 2). Also, an *in situ* calibration was conducted in the index-matching fluid. Matching the refractive index of the liquid with the plastic was key to minimize optical distortions due to wall curvature, and to obtain clear particle images for PIV post-processing. All particle images were dewarped into physical XY coordinates, which can not only correct varying magnifications in the field of view, ensuring a spatially coinciding interrogation volume from both views, but also verify the accuracy of calibration and compensate for possible errors associated with the misalignment of laser light sheet to the target slices. In addition, the transient pulsatile flow rates were measured and compared right before and after the PIV tests to ensure the stability of the flow system.

For PIV measurements in the ICA (see Figure 3A), three representative time instants (e.g., t = 0.03775, 0.11225, and 0.27530 s) were selected, and a total of 1,388 pairs of images were recorded for each time instant to calculate velocity vectors and blood flow rate, respectively. Similarly, for the tests in the ICASA sac (see Figure 3B), three designated time instants (i.e., t = 0.0722, 0.1901, and 0.3102 s) were employed, and the same number of images were obtained, separately. The Direct Correlator scheme in Insight 4G™ (TSI Inc., MN, United States) was employed for the post-processing of PIV images. Vectors were calculated within multiple window-screenings from 32 × 32-pixel with a 50% overlap to 16 × 16-pixel with a 50% overlap adaptively. The finial spatial resolution is ∼5 vector/mm. Subsequently, velocity vectors were phase-averaged over the collected 1,388 cycles to produce velocity vector distributions for each acquired phase of the cardiac cycle using an in-house C++ code. The uncertainty in the velocity measurements is estimated to be less than 2% of the magnitude. To quantify fluctuations at each phase, the normalized velocity magnitude at each point was calculated, from which the phase-averaged velocity and relative errors were calculated.

## 3 Numerical methodology

### 3.1 Geometry and mesh

**FIGURE 4:**
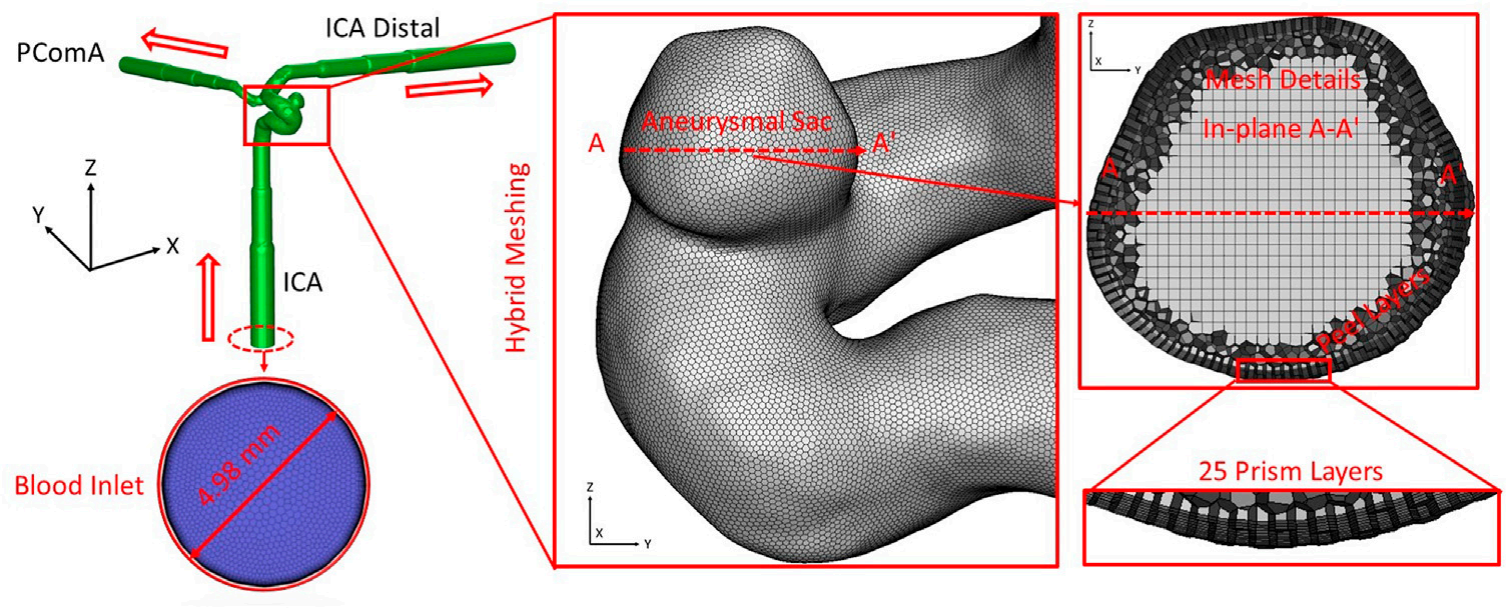
Schematic of the computational domain with hybrid mesh details in the ICASA model.

One patient-specific cerebral aneurysm model (see Figure 4) was built based on medical data (70–80 years old, female) provided by a hospital (Dayton, Ohio, United States). In the ICASA model, the blood flows in through the ICA and flows out from the bifurcated distal arteries, i.e., ICA distal and posterior communicating artery (PComA). Poly-hexcore meshing strategies were adopted to generate meshes for the ICASA model using ANSYS Fluent Meshing 2021 R2 (Ansys Inc., Canonsburg, PA, United States), and the mesh independence sensitivity was investigated and reported in the previous publication (Yi et al., 2022a). The final mesh has 2,741,603 elements with 25 prism layers, 3 peel layers, and size growth rate 1.05. Moreover, 25 near-wall prism layers in the finally selected mesh were generated and refined to guarantee the thickness of the first prism layer satisfying y+ < 1 using the flat plate boundary layer theory, where y+ is the dimensionless wall distance (Menter, 1994; Gomez-Miguel, 2005; Menter et al., 2006).

### 3.2 Governing equations

Due to the sensitivity to initial conditions and global hydrodynamic instability, it has been found that the physiologic pulsatile blood flow is turbulent even under a relatively small mean Reynolds number in both studies *in vitro* and *in silico* (Valen-Sendstad et al., 2011; Yagi et al., 2013; Jain et al., 2016; Saqr et al., 2020; Tupin et al., 2020; Yi et al., 2022b). Thus, the continuity and momentum equations can be written in tensor form, i.e.,

$$\frac{\partial u_{i}}{\partial x_{i}}=0,$$ (1)

$$\rho \frac{\partial u_{i}}{\partial t}+\rho \frac{\partial \left(u_{j}u_{i}\right)}{\partial x_{j}}=-\frac{\partial p}{\partial x_{i}}+\frac{\partial }{\partial x_{j}}\left[\left(\mu +\mu_{t}\right)\left(\frac{\partial u_{i}}{\partial x_{j}}+\frac{\partial u_{j}}{\partial x_{i}}\right)\right]+\rho g_{i},$$ (2)

where $u_{j}$ represents the blood flow velocity, *p* is the pressure, $g_{j}$ is the gravity, μ is the blood dynamic viscosity which was set as $2.0\times 10^{-3}\;Pa\cdot s$ in accordance with the PIV tests, and $\mu_{t}$ is the turbulent viscosity. With the transient pulsatile flow, the blood flow regime in the ICASA model is laminar-to-turbulence transitional flow. Therefore, the k−ω shear stress transport (SST) turbulence model (Menter, 1994) was adapted for this study, predicting the “laminar-to-turbulent” transition onset. In this study, the flow regime is assumed as incompressible and Newtonian, which has been widely employed in the previous study (Yu et al., 2019; Yi et al., 2022a).

### 3.3 Boundary and initial conditions

To validate CFD simulation results with *in vitro* PIV measurements, two transient pulsatile flow waveforms (see red colored curve in Figure 3) for the ICA inlet and another two corresponding waveforms (see blue colored curve in Figure 3) for the ICA distal outlet were employed for the CFD simulations, respectively, which were in accordance with the PIV settings. Additionally, the arterial walls are assumed to be stationary and non-slip, and the backflow direction at the ICA distal and PComA outlets were determined based on the known flow direction in the cell layer adjacent to ICA distal and PComA outlet.

### 3.4 Numerical settings

CFD modeling was proceeded using ANSYS Fluent 2021 R2 (Ansys Inc., Canonsburg, PA, United States). Simulation tasks were performed on a local HP Z840 workstation (Intel^®^ Xeon^®^ Processor E5-2687W v4 with dual processors, 24 cores, 48 threads, and 128 GB RAM). Under the designated time step size 5 × 10^−4^ s, it required ∼25 and ∼28 h to finish the simulation for one pulsatile period, i.e., T = 0.969 s and T = 0.995 s, respectively. Three cardiac periods were simulated for each modeling, and the results were analyzed based on the third period. The Semi-Implicit Method for Pressure Linked Equations (SIMPLE) algorithm was employed for the pressure-velocity coupling, and the least-squares cell-based scheme was applied to calculate the cell gradient. The second order scheme was used for pressure discretization. In addition, the second-order upwind scheme was applied for the discretization of momentum, turbulent kinetic energy, and specific dissipation rate. Convergence is registered for computing continuity, momentum, and supplementary equations when residuals are lower than 1.0 × 10^−3^.

## 4 Results and discussion

To validate the CFD model, the blood flow patterns (i.e., magnitude of velocity, normalized magnitude of velocity, velocity vector distribution, volumetric flow rate, and flow streamlines) were compared between *in vitro* PIV measurements and *in silico* CFD simulations for selected time instants within a cardiac cycle in ICASA model. Specifically, two designated planes, i.e., plane A and plane B (see Label 17 in Figures 2, 3A, B) in ICASA were employed to visualize the comparisons between PIV and CFD, respectively. One slice, i.e., plane A at X = 0.0726 m (according to coordinates in CFD model) crossing the ICA with three representative time instants (e.g., t = 0.03775, 0.11225, and 0.27530 s) were subtracted to investigate the flow characteristics near the aneurysmal sac, which aims to inspect flow field and volumetric flowrates approaching the aneurysmal sac. With three preferred time instants (i.e., t = 0.0722, 0.1901, and 0.3102 s), another slice, plane B, located at Y ≈ −0.5390 m (according to coordinates in CFD model) across the aneurysm sac in the middle was used to compare the flow features between CFD and PIV. In accordance with the experimental settings (see Figure 3), the periodic pulsatile flowrate waveforms with T = 0.969 s and T = 0.995 s were used in the *in silico* simulations for model validation, respectively. The two transitional pulsatile blood waveforms were generated by averaging 70 cardiac cycles periodically in experiments (see Figure 3).

**FIGURE 5:**
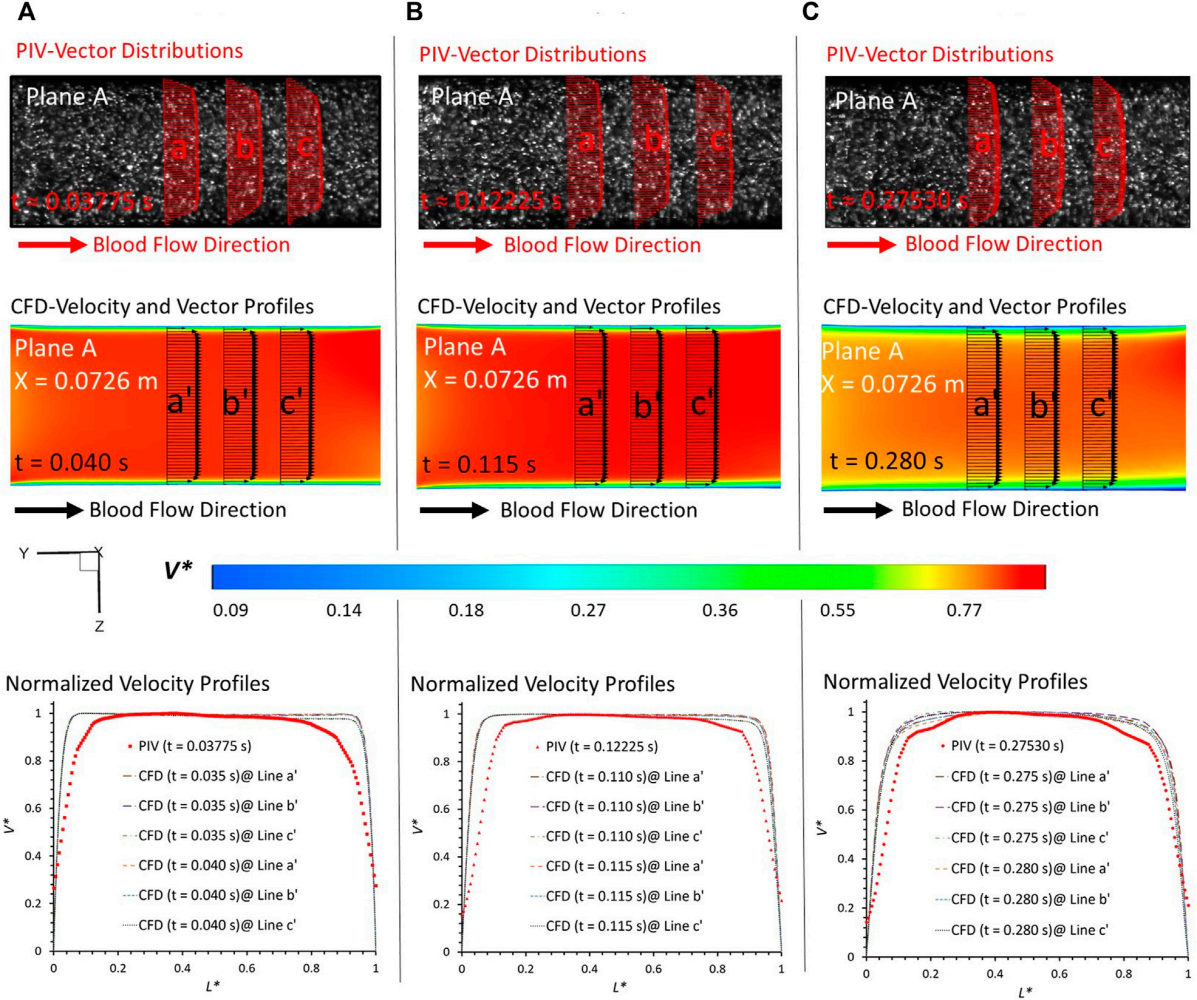
Comparisons at plane A at X = 0.0726 m (according to the coordinates in CFD) between PIV measurements and CFD simulations at designated time instants: **(A)** t = 0.03775 s, **(B)** t = 0.11225 s, and **(C)** t = 0.27530 s.

**FIGURE 6:**
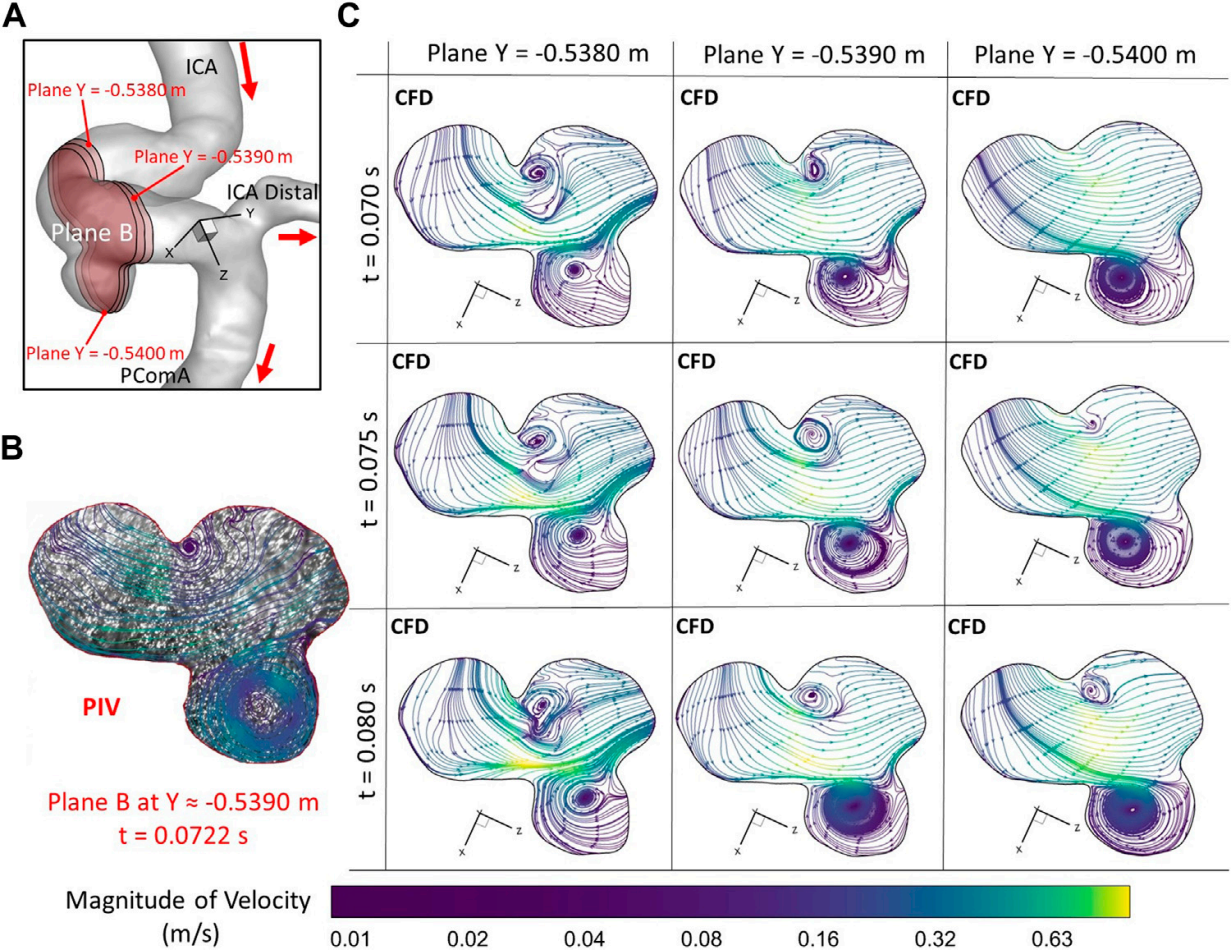
Comparisons of flow streamlines and magnitudes of velocity at the extracted plane B (i.e., plane Y ≈ −0.5390 m according to the coordinates in CFD) between PIV measurements and CFD simulations at the time instant t = 0.078 s: **(A)** Schematic planes for the comparisons between PIV and CFD. **(B)** Distributions of flow streamlines and magnitudes of velocity in PIV. **(C)** Distributions of flow streamlines and magnitudes of velocity in CFD.

**FIGURE 7:**
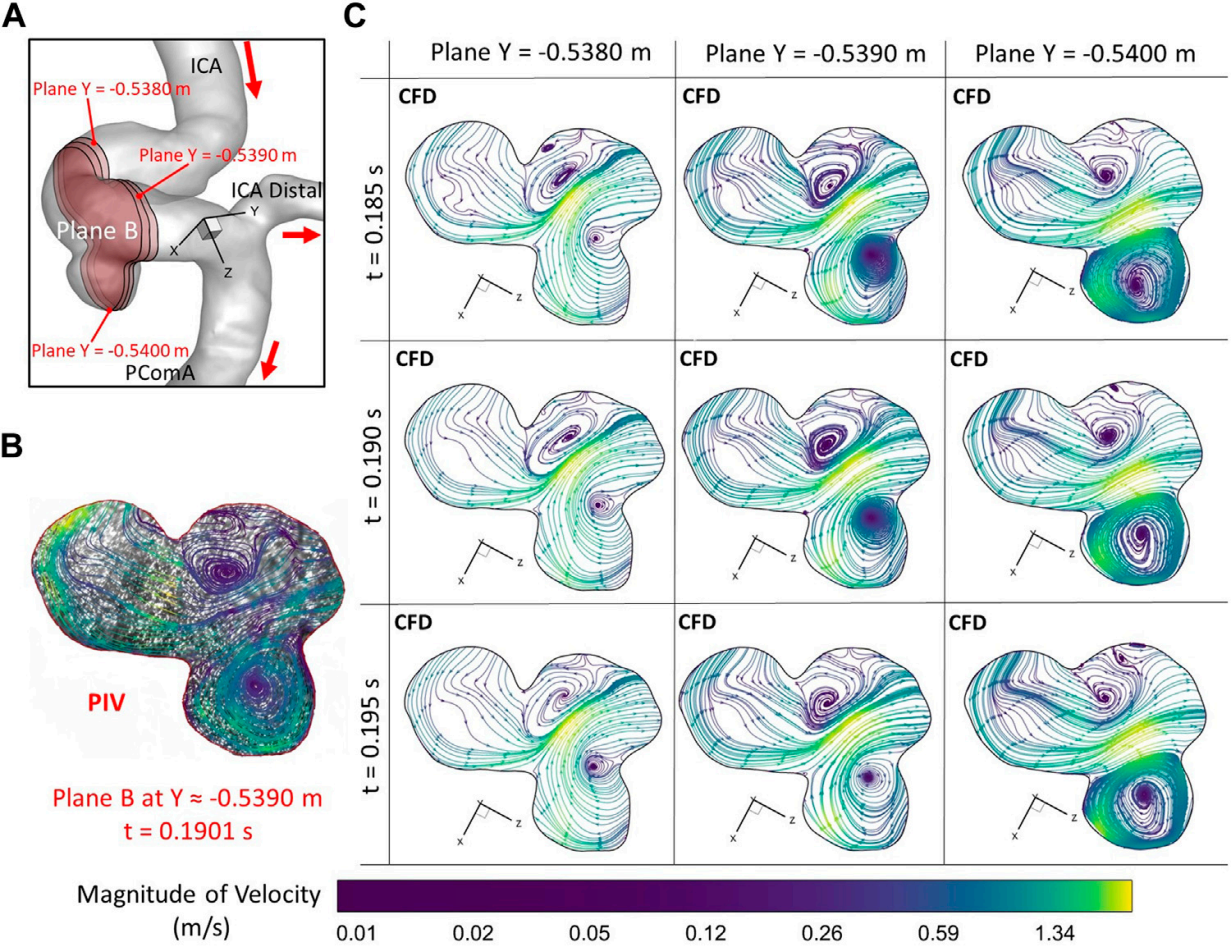
Comparisons of flow streamlines and magnitudes of velocity at the extracted plane B (i.e., plane Y ≈ −0.5390 m according to the coordinates in CFD) between PIV measurements and CFD simulations at the time instant t = 0.1901 s: **(A)** Schematic planes for the comparisons between PIV and CFD. **(B)** Distributions of flow streamlines and magnitudes of velocity in PIV. **(C)** Distributions of flow streamlines and magnitudes of velocity in CFD.

**FIGURE 8:**
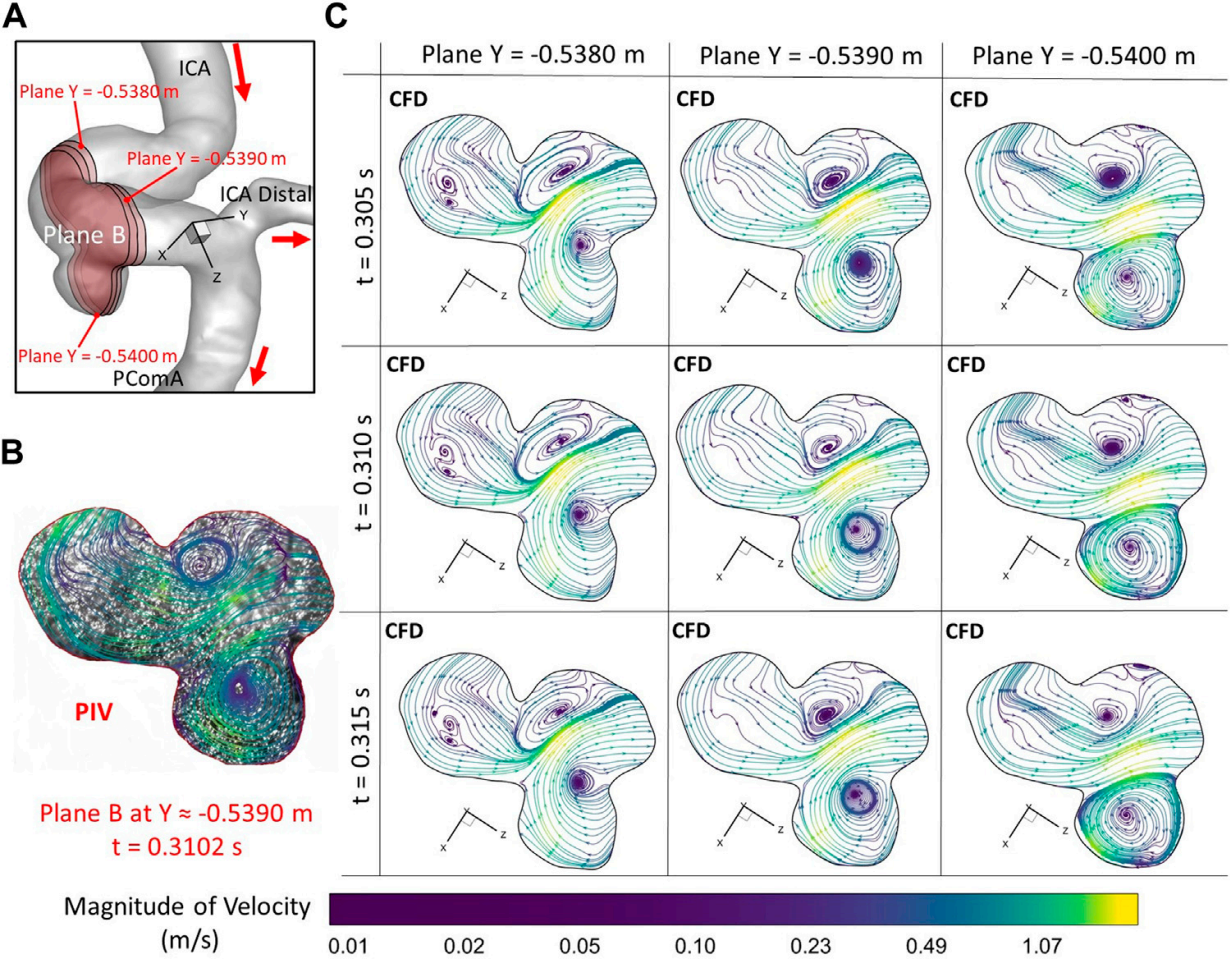
Comparisons of flow streamlines and magnitudes of velocity at the extracted plane B (i.e., plane Y ≈ −0.5390 m according to the coordinates in CFD) between PIV measurements and CFD simulations at the time instant t = 0.3102 s: **(A)** Schematic planes for the comparisons between PIV and CFD. **(B)** Distributions of flow streamlines and magnitudes of velocity in PIV. **(C)** Distributions of flow streamlines and magnitudes of velocity in CFD.

**FIGURE 9:**
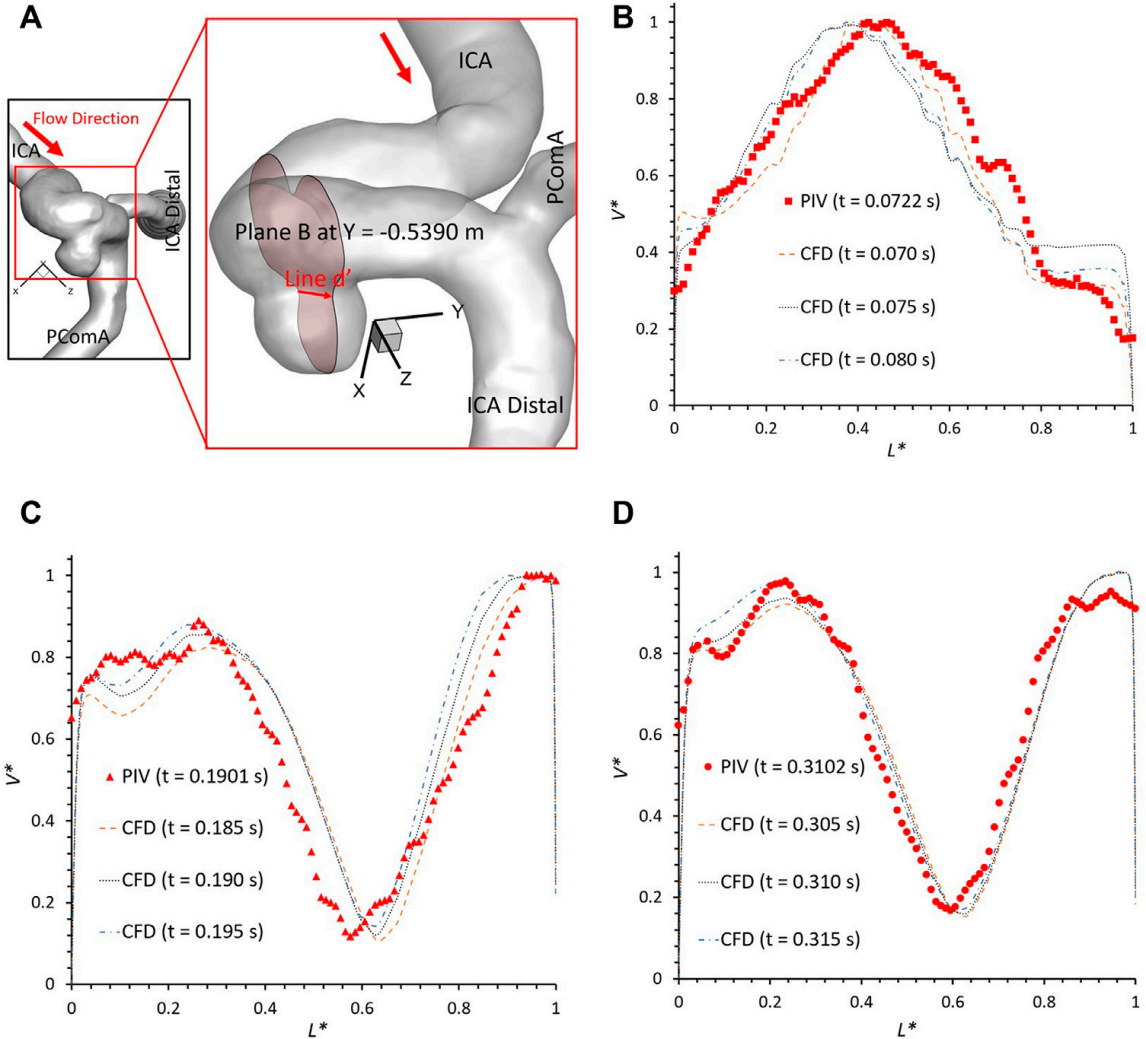
Velocity profile comparisons at line d’ of the plane B (i.e., plane Y ≈ −0.5390 m according to the coordinates in CFD) between PIV measurements and CFD simulations at different time instants: **(A)** schematic line d’ for comparisons, **(B)** t = 0.0722 s, **(C)** t = 0.1901 s, and **(D)** t = 0.3102 s.

Dependent variables employed in the model validation (see Tables 1, 2; Figures 5–9) were defined as follows. In the PIV tests, the ICA diameter close to the aneurysm is defined by

$$D_{ICA}=\frac{l_{a}+l_{b}+l_{c}}{3},$$ (3)

where $l_{a}$ , $l_{b}$ , and $l_{c}$ are the lengths of line a, b, and c, respectively. The blood volumetric flow rate V in PIV tests is calculated using

$$V=\frac{V_{a}+V_{b}+V_{c}}{3},$$ (4)

$$V_{a}=\oint v_{a}ds,$$ (5)

$$V_{b}=\oint v_{b}ds,$$ (6)

$$V_{c}=\oint v_{c}ds,$$ (7)

where $V_{a}$ , $V_{b}$ , and $V_{c}$ are the volumetric flowrates in the cross-section through line a, b, and c in plane A, respectively. $v_{a}$ , $v_{b}$ , and $v_{c}$ are velocity magnitudes in line a, b, and c, respectively. s is the differential area perpendicularly across plane A with corresponded local velocities (i.e., $v_{a}$ , $v_{b}$ , and $v_{c}$ ). In contrast, the blood flow rate V in the CFD simulations is obtained directly from the periodic pulsatile flowrate profiles (see Figure 3) at the corresponding instants. The normalized blood velocity $V^{*}$ in CFD at plane A is defined as

$$V^{*}=\frac{{\left(v_{y}^{2}+v_{z}^{2}\right)}^{1/2}}{{\left(v_{y}^{2}+v_{z}^{2}\right)}_{\mathrm{Max}.}^{1/2}},$$ (8)

where $v_{y}$ and $v_{z}$ are the blood velocity magnitudes in CFD simulations in the Y and Z directions, respectively. ${\left({v_{y}}^{2}+{v_{z}}^{2}\right)}_{\mathrm{Max}.}^{1/2}$ is the maximum magnitude of velocity at plane A. Also, the normalized blood velocity V* in CFD at plane B is defined as

$$V^{*}=\frac{{\left(v_{x}^{2}+v_{z}^{2}\right)}^{1/2}}{{\left(v_{x}^{2}+v_{z}^{2}\right)}_{\mathrm{Max}.}^{1/2}},$$ (9)

where $v_{x}$ and $v_{z}$ are the blood velocity magnitudes of CFD simulations in the directions of X and Z, respectively. ${\left(v_{x}^{2}+v_{z}^{2}\right)}_{\mathrm{Max}.}^{1/2}$ is the maximum magnitude of velocity at plane B. It needs to mention that Cartesian YZ/XZ coordinate system in CFD corresponds to Cartesian XY coordinate system in two separate PIV measurements. Thus, the normalized blood velocity $V^{*}$ in PIV can be defined as

$$V^{*}=\frac{{\left(v_{x}^{\prime 2}+v_{y}^{\prime 2}\right)}^{1/2}}{{\left(v_{x}^{\prime 2}+v_{y}^{\prime 2}\right)}_{\mathrm{Max}.}^{1/2}},$$ (10)

where $v_{x}^{\prime }$ and $v_{y}^{\prime }$ are the flow velocity magnitudes in PIV measurements in X direction and Y direction, respectively. ${\left(v_{x}^{\prime 2}+v_{y}^{\prime 2}\right)}_{\mathrm{Max}.}^{1/2}$ is the maximum magnitude of velocity in the PIV measured flow field.

**TABLE 1:** Volumetric flowrate *V* (m^3^/s) comparisons among flowmeter, CFD and PIV under selected time instants.

	Flowmeter	CFD	PIV	Relative difference (%) between	
				Flowmeter and CFD	Flowmeter and PIV
ICA diameter $D_{ICA}$ (m)	Not applicable	1.034e-2	1.037e-2	Not applicable	Not applicable
t = 0.038 s	3.04548e-5	3.05550e-5	2.82509e-5	0.33	7.24
t = 0.112 s	8.24484e-5	8.25486e-5	7.62372e-5	0.12	7.53
t = 0.275 s	6.23122e-5	6.24123e-5	5.92066e-5	0.16	4.98

**TABLE 2:** Comparisons in averaged V* between PIV measurements and CFD simulations under selected time instants.

	CFD	PIV	Relative errors (%)
Length of Line *d’* (m)	8.62e-3	8.13e-3	6.03
t = 0.072 s	0.69	0.63	9.52
t = 0.190 s	0.63	0.62	1.59
t = 0.310 s	0.69	0.68	1.47

In PIV measurements, the velocity is estimated between two instants within a short time duration. To eliminate the errors from the time delay between the two images (image A and image B) in PIV, the CFD results extracted from two neighboring instants with a time gap of 0.005 s were employed to evaluate the agreements with the corresponding PIV data at a specific time instant. For example, for the PIV tests at t = 0.03775 s, the results at t = 0.035 s and t = 0.040 s were extracted from the CFD simulations. From the visualized velocity vectors and normalized magnitude of velocity V* on the line a, b, c, a’, b’, and c’ of plane A at all three representative time instants (i.e., t = 0.03775 s, t = 0.11225 s, and t = 0.27530 s) (see Figure 5), it can be found that blood flow patterns approaching the aneurysmal sac match well in the comparison of PIV measurements and CFD simulations when L* ranges from 0.1 to 0.9. A good agreement can also be observed from the comparisons in volumetric flowrates between flowmeter and CFD, with relative differences smaller than 0.33% at t = 0.038 s (see Table 1). Similar comparisons can be found between the flowmeter and PIV, following a relative difference smaller than 7.53% at t = 0.112 s (see Table 1). It is interesting to discover that such comparable results of blood volumetric flow rates indicate that the flowmeter system (see Section 2.2) performs well in measuring the transient pulsatile flows.

To further consolidate the model validation, the locations of flow vortex in the aneurysmal sac which was visualized by flow streamlines have been compared between CFD simulations and PIV measurements in the extracted plane B (i.e., Y ≈ −0.5390 m) at three representative time instants (e.g., t = 0.0722 s, t = 0.1901 s, and t = 0.3102 s) during a cardiac cycle, shown in Figures 6–8. It is worth mentioning that the simulation results at three neighboring time instants with a time gap of 0.005 s were extracted and compared to corresponding visualized flow streamlines in PIV tests at a specific time instant (see Figures 6–8), which is similar to the comparisons of flow patterns in the ICA between CFD and PIV. Additionally, the influences by the laser thickness (i.e., 2 mm) on the designated slice position and duration-based nature of the PIV system (e.g., the time delay between image A and image B) need to be evaluated to eliminate the errors. Thus, three extracted slices in the ICASA model (e.g., plane Y = −0.5380 m, −0.5390 m, −0.5400 m) in CFD simulations were adopted to compare PIV tests in the extracted plane B. Figures 6–8 present that CFD simulations can predict the vortex locations to match the PIV results essentially at all studied time instants. In both *in vitro* and *in silico* studies, the vortex travels from aneurysmal sac center at t = 0.0722 s to the upper right corner of the aneurysmal sac at t = 0.3102 s in the extracted plane B of the ICASA model. The comparisons between CFD and PIV in normalized velocity magnitude V* on the in-plane line d’ were shown in Figure 9, in which the velocity profile values on the line d’ were extracted from the in-model plane Y = -0.5390 m. It can be observed that the small relative errors (<9.52%) of averaged V* (see Table 2) also proved that CFD simulation can capture the flow patterns as presented in the experimental investigations.

However, there are small differences between CFD simulations and PIV measurements in some local regions, mainly in the near wall regions (i.e., 0 < L* < 0.1 and 0.9 < L* < 1.0), which were also mentioned in previous studies (Raschi et al., 2012). Such differences can be caused by several factors, i.e., 1) the final physical *in vitro* model produced from the STL data using SolidWorks (Dassault Systèmes, Vélizy-Villacoublay, France) can have slight geometric differences with the original STL data that has the potential to influence the flow results significantly (see Section 2.1) as the CFD simulation is sensitive to the wall configurations; 2) the light reflection and refraction effects from the internal wall of the physical model contribute to image noises that can affect the identifications of fluorescent polyethylene particles trajectory especially near the boundary; 3) the complicated curvature of the wall of the ICASA model disturbs the lights into the camera and then generate blurring spots on the PIV images; 4) the cycle-to-cycle fluctuations in the pulsatile flow affect the accuracy of phase averaged results in the PIV tests, and then contribute to the differences when comparing to the CFD simulations; 5) a heart rate mimicked by pulsatile blood pump with a confidence interval of 60 ± 0.5 beats per minute may cause the difference between CFD and PIV when deciding the numerical modeling boundary conditions (see Figure 2); and 6) the minor error in the perspective angle (e.g., ∼90°) between the laser sheet and the camera axis, the thickness of laser sheet (e.g., ∼2 mm), could affect the location of the extracted slice in the ICASA model, which are more apparent in the velocity profile comparisons at line d’ (see Figure 9).

The current study still has limitations in the clinical analysis of hemodynamic characteristic in the CAs. Specifically, the employed *in vitro* ICASA model with the scaling factor of 3.0 and the studied blood analogue fluid (i.e., water with seeded fluorescent polyethylene particles) in the experiments may provide a more qualitative analysis rather than a quantitative hemodynamic investigation in our current work. Blood is a non-Newtonian fluid with shear-thinning features that was also not considered in this study, as well as that deforming interactions between blood and arterial walls still need be identified explicitly. Our following research will model the hemodynamic characteristics in the cerebral aneurysm model *in vitro* and *in silico*, integrating with interactions between the deformations of arterial walls and cardiac pulsatile blood flow field, by using a two-way fluid-structure interaction manner (Samaee et al., 2022).

## 5 Conclusion

Using both experimental PIV measurements and CFD simulations, an *in vitro* validated *in silico* ICASA model for the simplified Newtonian flow was developed which can be used to investigate the hemodynamic factors such as WSS and OSI that could influence the initiation, progression, and rupture of CAs, by integrating CFD with statistical analysis for a large amount of patient-specific cases in the future. Despite those tremendous efforts have been spent on optimizing the simulation and the PIV experiment to eliminate possible errors, it is almost impossible to reach a perfect alignment between the PIV and CFD simulation results. Decent agreements were found between *in vitro* PIV tests and *in silico* CFD investigations in the blood flow rate, normalized velocity profiles, flow streamlines, and vortex locations, which enables the developed CFD model to investigate hemodynamic factors on the pathophysiology of CAs. Also, the employed PIV measurements and CFD modeling in this study provide a pathway for other researchers to build an experimentally validated numerical model, which has the capability to accurately investigate hemodynamics associated with cardiovascular and neurovascular diseases.

## Data Availability

The raw data supporting the conclusion of this article will be made available by the authors, without undue reservation.
